# The genome sequence of the Six-belted Clearwing, *Bembecia ichneumoniformis *(Denis & Schiffermüller, 1775)

**DOI:** 10.12688/wellcomeopenres.20279.1

**Published:** 2023-11-10

**Authors:** Douglas Boyes, University of Oxford and Wytham Woods Genome Acquisition Lab, Darwin Tree of Life Barcoding collective, Wellcome Sanger Institute Tree of Life programme, Wellcome Sanger Institute Scientific Operations: DNA Pipelines collective, Tree of Life Core Informatics collective, Darwin Tree of Life Consortium

**Affiliations:** 1 UK Centre for Ecology & Hydrology, Wallingford, England, UK

**Keywords:** Bembecia ichneumoniformis, Six-belted Clearwing, genome sequence, chromosomal, Lepidoptera

## Abstract

We present a genome assembly from an individual male *Bembecia ichneumoniformis* (the Six-belted Clearwing; Arthropoda; Insecta; Lepidoptera; Sesiidae). The genome sequence is 511.4 megabases in span. Most of the assembly is scaffolded into 31 chromosomal pseudomolecules, including the Z sex chromosome. The mitochondrial genome has also been assembled and is 15.32 kilobases in length. Gene annotation of this assembly on Ensembl identified 12,114 protein coding genes.

## Species taxonomy

Eukaryota; Metazoa; Eumetazoa; Bilateria; Protostomia; Ecdysozoa; Panarthropoda; Arthropoda; Mandibulata; Pancrustacea; Hexapoda; Insecta; Dicondylia; Pterygota; Neoptera; Endopterygota; Amphiesmenoptera; Lepidoptera; Glossata; Neolepidoptera; Heteroneura; Ditrysia; Apoditrysia; Sesioidea; Sesiidae; Sesiinae; Synanthedonini; *Bembecia*; *Bembecia ichneumoniformis* (Denis & Schiffermüller, 1775) (NCBI:txid301037).

## Background

The Six-belted Clearwing ( *Bembecia ichneumoniformis*) is a member of the Sesiidae family of clearwing moths. Thought to be Batesian mimics of Hymenoptera, Clearwing moths have narrow wings with transparent regions free of scales, and black bodies banded with red or yellow. The specific name *ichneumoniformis* means that its shape and colours, as well as the structure of its wings, evokes certain ichneumonids. Males of *Bembecia ichneumoniformis* exhibit six yellow bands across the abdomen, while females have five. In both sexes, there are orange scales at the tip and along the central bar of the forewings, with legs that are largely yellow ( Waring *et al.*, 2017).

This species has been recorded in most of Europe and Asia Minor, the Caucasus, northern Iran and the Near East ( GBIF Secretariat, 2023). In Britain and Ireland, the moth is classified as “Nationally Scarce B” ( Butterfly Conservation, 2023), and is recorded mostly from England and Wales. The moth favours habitats such as chalk and coastal grasslands featuring grassy swards, as well as rough upland fields, embankments, quarries, and cliffs, avoiding areas with heavy grazing ( Waring *et al.*, 2017).

The caterpillars feed on specific plants including common bird’s-foot-trefoil ( *Lotus corniculatus*), kidney vetch ( *Anthyllis vulneraria*), and possibly horseshoe vetch ( *Hippocrepis comosa*). Indicators of larval presence, such as frass, may be found along the main root of the larval food plant ( Waring *et al.*, 2017). Adult specimens are often collected using sweep nets. The larval feeding period extends from July until the following May, and the species overwinters as larvae. The flight season occurs once a year, usually from late June to mid-August. During this period, males are particularly attracted to pheromone lures.

The genome of the six-belted clearwing, *Bembecia ichneumoniformis*, was sequenced as part of the Darwin Tree of Life Project, a collaborative effort to sequence all named eukaryotic species in the Atlantic Archipelago of Britain and Ireland. Here we present a chromosomally complete genome sequence for *Bembecia ichneumoniformis*, based on one male specimen from Wytham Woods, Oxfordshire, UK.

## Genome sequence report

**Figure 1.**
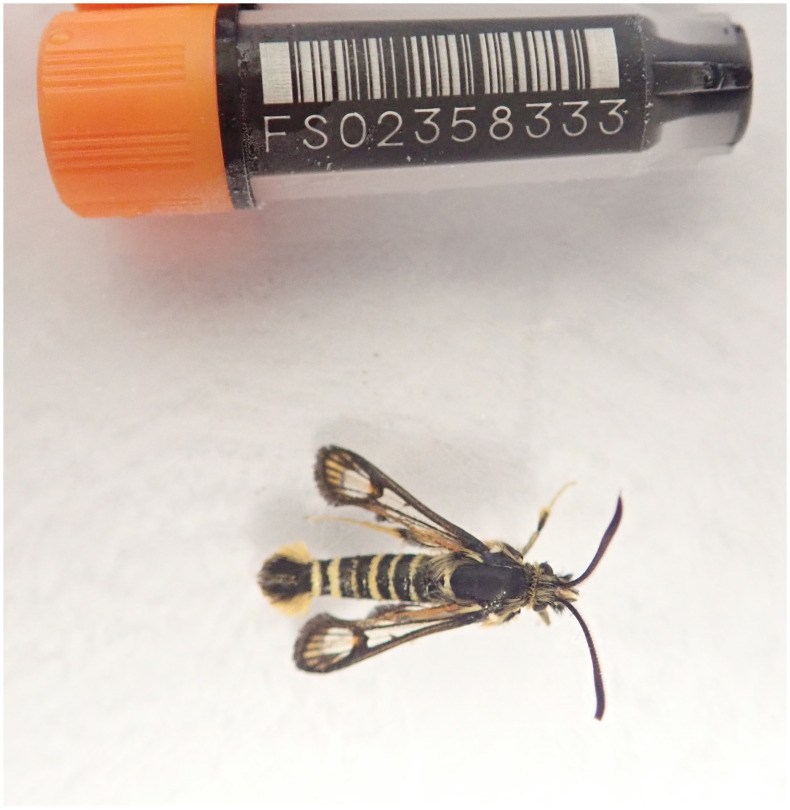
Photograph of the *Bembecia ichneumoniformis* (ilBemIchn1) specimen used for genome sequencing.

The genome was sequenced from one male *Bembecia ichneumoniformis* ( Figure 1) collected from Wytham Woods, Oxfordshire, UK (51.77, –1.33). A total of 30-fold coverage in Pacific Biosciences single-molecule HiFi long reads and 93-fold coverage in 10X Genomics read clouds were generated. Primary assembly contigs were scaffolded with chromosome conformation Hi-C data. Manual assembly curation corrected 33 missing joins or mis-joins and removed one haplotypic duplication, reducing the scaffold number by 32.76% and increasing the scaffold N50 by 6.43%.

**Figure 2.**
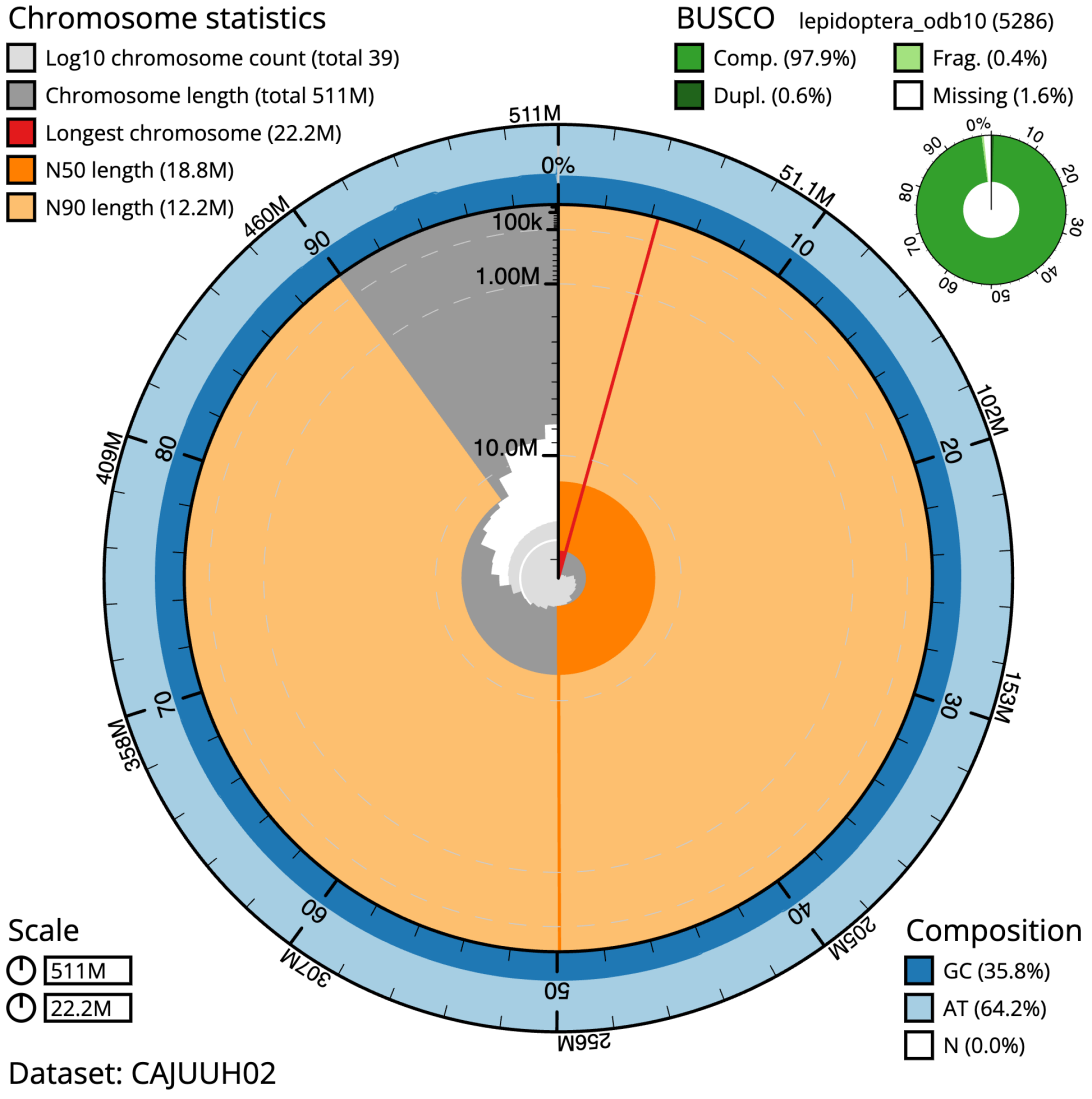
Genome assembly of *Bembecia ichneumoniformis*, ilBemIchn1.2: metrics. The BlobToolKit Snailplot shows N50 metrics and BUSCO gene completeness. The main plot is divided into 1,000 size-ordered bins around the circumference with each bin representing 0.1% of the 511,410,357 bp assembly. The distribution of scaffold lengths is shown in dark grey with the plot radius scaled to the longest scaffold present in the assembly (22,154,014 bp, shown in red). Orange and pale-orange arcs show the N50 and N90 scaffold lengths (18,823,830 and 12,162,325 bp), respectively. The pale grey spiral shows the cumulative scaffold count on a log scale with white scale lines showing successive orders of magnitude. The blue and pale-blue area around the outside of the plot shows the distribution of GC, AT and N percentages in the same bins as the inner plot. A summary of complete, fragmented, duplicated and missing BUSCO genes in the lepidoptera_odb10 set is shown in the top right. An interactive version of this figure is available at https://blobtoolkit.genomehubs.org/view/ilBemIchn1.2/dataset/CAJUUH02/snail.

**Figure 3.**
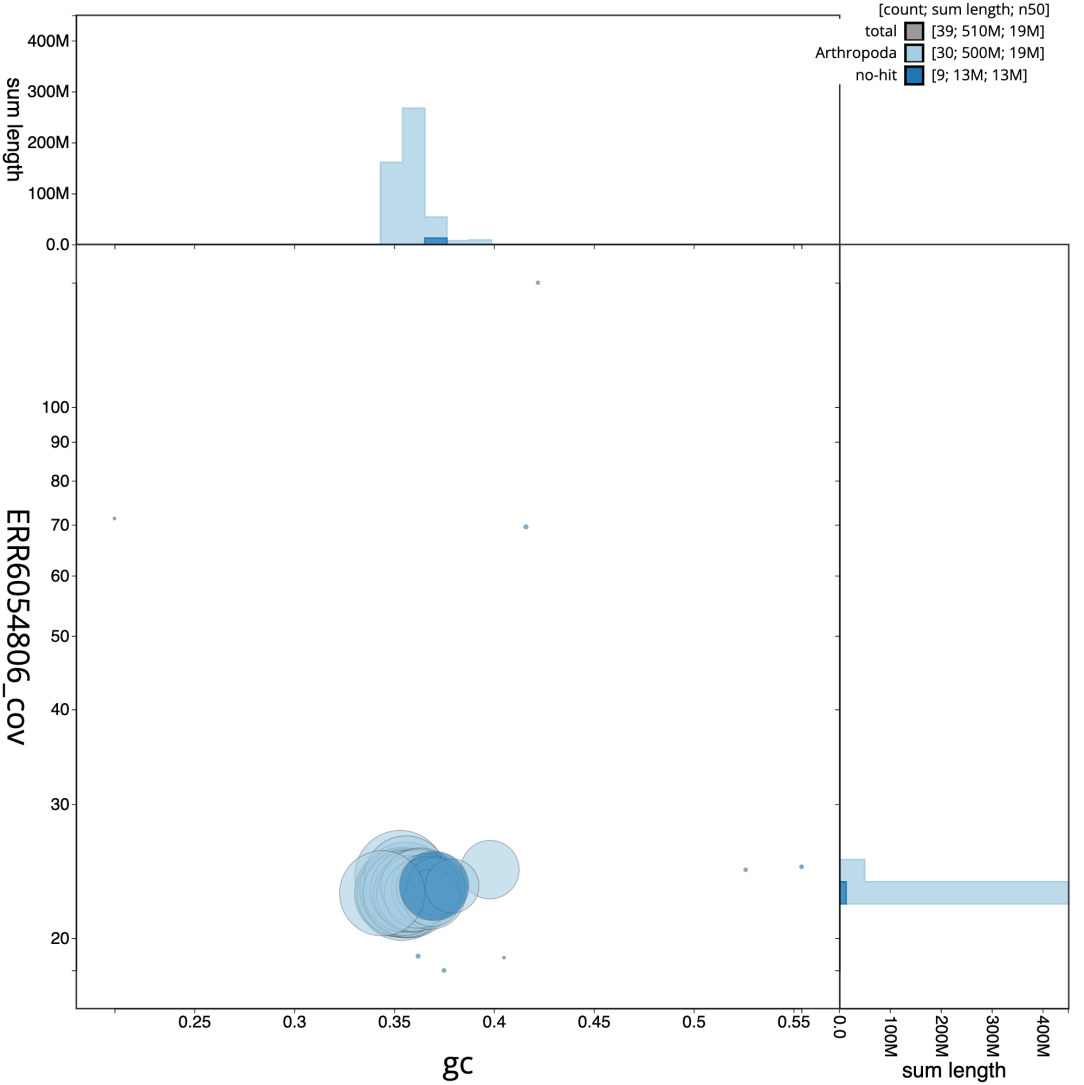
Genome assembly of *Bembecia ichneumoniformis*, ilBemIchn1.2: BlobToolKit GC-coverage plot. Scaffolds are coloured by phylum. Circles are sized in proportion to scaffold length. Histograms show the distribution of scaffold length sum along each axis. An interactive version of this figure is available at https://blobtoolkit.genomehubs.org/view/ilBemIchn1.2/dataset/CAJUUH02/blob.

**Figure 4.**
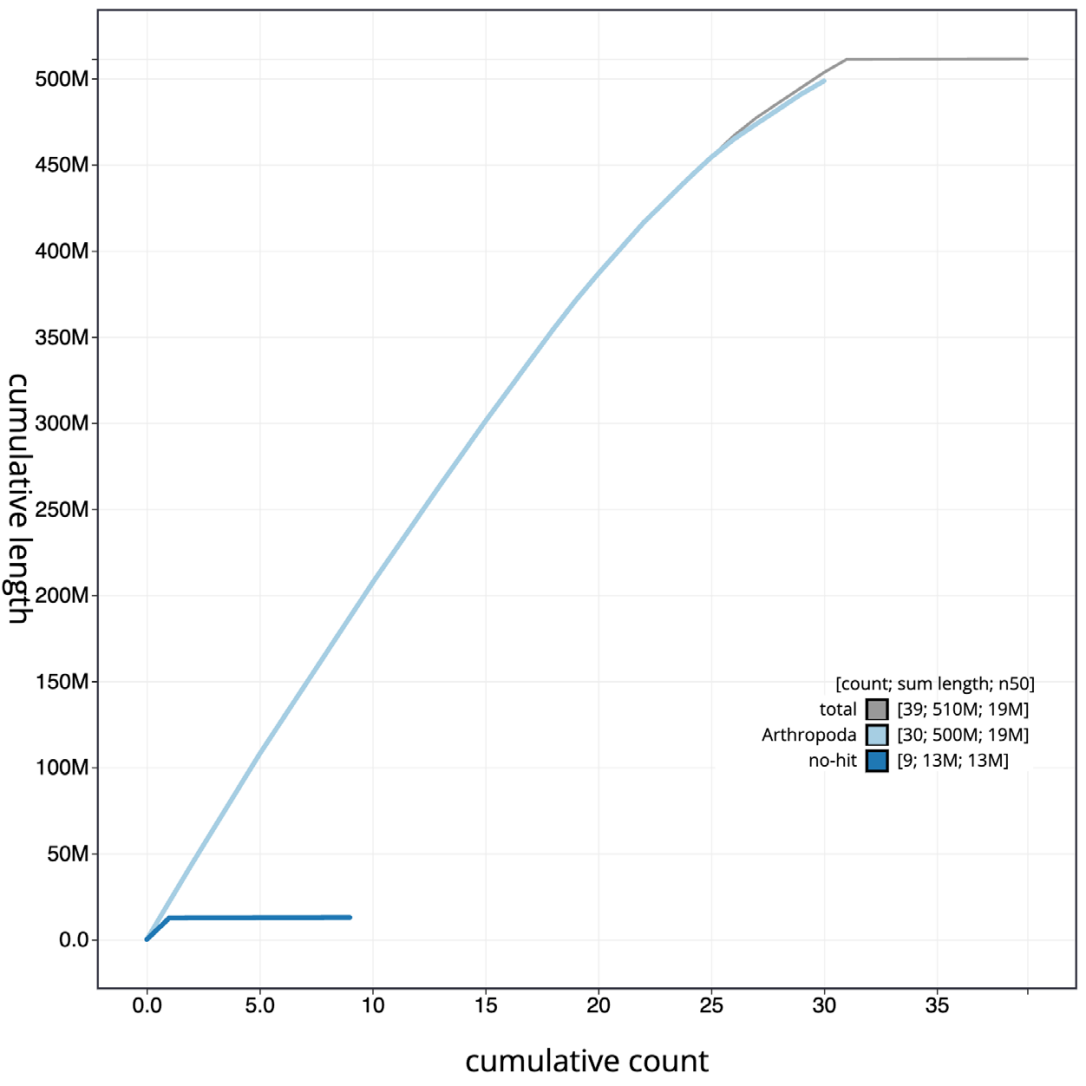
Genome assembly of *Bembecia ichneumoniformis*, ilBemIchn1.2: BlobToolKit cumulative sequence plot. The grey line shows cumulative length for all scaffolds. Coloured lines show cumulative lengths of scaffolds assigned to each phylum using the buscogenes taxrule. An interactive version of this figure is available at https://blobtoolkit.genomehubs.org/view/ilBemIchn1.2/dataset/CAJUUH02/cumulative.

**Figure 5.**
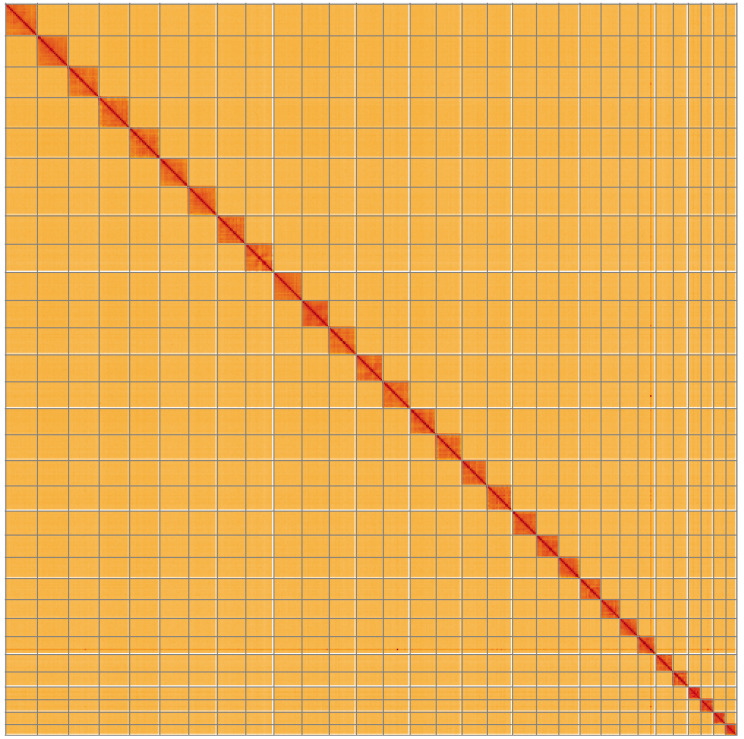
Genome assembly of *Bembecia ichneumoniformis*, ilBemIchn1.2: Hi-C contact map of the ilBemIchn1.2 assembly, visualised using HiGlass. Chromosomes are shown in order of size from left to right and top to bottom. An interactive version of this figure may be viewed at https://genome-note-higlass.tol.sanger.ac.uk/l/?d=CTGaP8xaT7CnLlRJWDRwrg.

The final assembly has a total length of 511.4 Mb in 38 sequence scaffolds with a scaffold N50 of 18.8 Mb ( Table 1). A summary of the assembly statistics is shown in Figure 2, while the distribution of assembly scaffolds on GC proportion and coverage is shown in Figure 3. The cumulative assembly plot in Figure 4 shows curves for subsets of scaffolds assigned to different phyla. Most (99.95%) of the assembly sequence was assigned to 31 chromosomal-level scaffolds, representing 30 autosomes and the Z sex chromosome. Chromosome-scale scaffolds confirmed by the Hi-C data are named in order of size ( Figure 5; Table 2). While not fully phased, the assembly deposited is of one haplotype. Contigs corresponding to the second haplotype have also been deposited. The mitochondrial genome was also assembled and can be found as a contig within the multifasta file of the genome submission.

**Table 1.** Genome data for *Bembecia ichneumoniformis*, ilBemIchn1.2.

Project accession data		
Assembly identifier	ilBemIchn1.2	
Species	*Bembecia ichneumoniformis*	
Specimen	ilBemIchn1	
NCBI taxonomy ID	301037	
BioProject	PRJEB45128	
BioSample ID	SAMEA7701282	
Isolate information	ilBemIchn1, male: abdomen (DNA sequencing), head and thorax (Hi-C data)	
Assembly metrics *		*Benchmark*
Consensus quality (QV)	57.8	*≥ 50*
*k*-mer completeness	99.99%	*≥ 95%*
BUSCO **	C:97.9%[S:97.4%,D:0.6%], F:0.4%,M:1.6%,n:5,286	*C ≥ 95%*
Percentage of assembly mapped to chromosomes	99.95%	*≥ 95%*
Sex chromosomes	Z chromosome	*localised homologous pairs*
Organelles	Mitochondrial genome assembled	*complete single alleles*
Raw data accessions		
PacificBiosciences SEQUEL II	ERR6454729	
10X Genomics Illumina	ERR6054807, ERR6054808, ERR6054809, ERR6054806	
Hi-C Illumina	ERR6054810	
Genome assembly		
Assembly accession	GCA_910589475.2	
*Accession of alternate* *haplotype*	GCA_910589565.1	
Span (Mb)	511.4	
Number of contigs	74	
Contig N50 length (Mb)	15.6	
Number of scaffolds	38	
Scaffold N50 length (Mb)	18.8	
Longest scaffold (Mb)	22.2	
Genome annotation		
Number of protein-coding genes	12,114	
Number of non-coding genes	2,916	
Number of gene transcripts	22,684	

* Assembly metric benchmarks are adapted from column VGP-2020 of “Table 1: Proposed standards and metrics for defining genome assembly quality” from ( Rhie *et al.*, 2021). ** BUSCO scores based on the lepidoptera_odb10 BUSCO set using v5.3.2. C = complete [S = single copy, D = duplicated], F = fragmented, M = missing, n = number of orthologues in comparison. A full set of BUSCO scores is available at https://blobtoolkit.genomehubs.org/view/ilBemIchn1.2/dataset/CAJUUH02/busco.

**Table 2.** Chromosomal pseudomolecules in the genome assembly of *Bembecia ichneumoniformis*, ilBemIchn1.

INSDC accession	Chromosome	Length (Mb)	GC%
OU342520.1	1	22.15	35.5
OU342521.1	2	21.74	35.5
OU342522.1	3	21.44	35.5
OU342523.1	4	21.33	35.5
OU342524.1	5	21.1	35.5
OU342525.1	6	20.21	35.5
OU342526.1	7	20.03	35.0
OU342527.1	8	19.84	35.0
OU342528.1	9	19.68	35.5
OU342529.1	10	19.63	36.0
OU342531.1	11	18.94	35.5
OU342532.1	12	18.82	35.5
OU342533.1	13	18.74	35.5
OU342534.1	14	18.27	35.5
OU342535.1	15	18.07	35.5
OU342536.1	16	17.69	35.5
OU342537.1	17	17.62	36.5
OU342538.1	18	16.76	36.0
OU342539.1	19	15.6	36.0
OU342540.1	20	14.81	36.0
OU342541.1	21	14.71	36.0
OU342542.1	22	13.17	37.0
OU342543.1	23	12.69	36.5
OU342544.1	24	12.55	37.0
OU342545.1	25	12.16	36.0
OU342546.1	26	10.48	36.5
OU342547.1	27	8.92	40.0
OU342548.1	28	8.77	37.0
OU342549.1	29	8.61	37.0
OU342550.1	30	7.59	38.0
OU342530.1	Z	19.09	34.5
OU342551.2	MT	0.02	21.5

The estimated Quality Value (QV) of the final assembly is 57.8 with *k*-mer completeness of 99.99%, and the assembly has a BUSCO v5.3.2 completeness of 97.9% (single = 97.4%, duplicated = 0.6%), using the lepidoptera_odb10 reference set ( *n* = 5,286).

Metadata for specimens, barcode results, spectra estimates, sequencing runs, contaminants and pre-curation assembly statistics can be found at https://links.tol.sanger.ac.uk/species/301037.

## Genome annotation report

The *Bembecia ichneumoniformis* genome assembly (GCA_910589475.1) was annotated using the Ensembl rapid annotation pipeline ( Table 1; https://rapid.ensembl.org/Bembecia_ichneumoniformis_GCA_910589475.1/Info/Index). The resulting annotation includes 22,684 transcribed mRNAs from 12,114 protein-coding and 2,916 non-coding genes.

## Methods

### Sample acquisition and nucleic acid extraction

A male *Bembecia ichneumoniformis* (specimen ID Ox000506, individual ilBemIchn1) was collected from Wytham Woods, Oxfordshire (biological vice-county Berkshire), UK (latitude 51.77, longitude –1.33) on 2020-06-23, using an API pheromone lure. The specimen was collected and identified by Douglas Boyes (University of Oxford) and preserved on dry ice.

DNA was extracted at the Tree of Life laboratory, Wellcome Sanger Institute (WSI). The ilBemIchn1 sample was weighed and dissected on dry ice with tissue set aside for Hi-C sequencing. Tissue from the abdomen disrupted using a Nippi Powermasher fitted with a BioMasher pestle. High molecular weight (HMW) DNA was extracted using the Qiagen MagAttract HMW DNA extraction kit. Low molecular weight DNA was removed from a 20 ng aliquot of extracted DNA using the 0.8X AMpure XP purification kit prior to 10X Chromium sequencing; a minimum of 50 ng DNA was submitted for 10X sequencing. HMW DNA was sheared into an average fragment size of 12–20 kb in a Megaruptor 3 system with speed setting 30. Sheared DNA was purified by solid-phase reversible immobilisation using AMPure PB beads with a 1.8X ratio of beads to sample to remove the shorter fragments and concentrate the DNA sample. The concentration of the sheared and purified DNA was assessed using a Nanodrop spectrophotometer and Qubit Fluorometer and Qubit dsDNA High Sensitivity Assay kit. Fragment size distribution was evaluated by running the sample on the FemtoPulse system.

Protocols used by the Tree of Life laboratory are publicly available on protocols.io: https://dx.doi.org/10.17504/protocols.io.8epv5xxy6g1b/v1.

### Sequencing

Pacific Biosciences HiFi circular consensus and 10X Genomics read cloud DNA sequencing libraries were constructed according to the manufacturers’ instructions. DNA sequencing was performed by the Scientific Operations core at the WSI on Pacific Biosciences SEQUEL II (HiFi) and Illumina NovaSeq 6000 (10X) instruments. Hi-C data were also generated from head and thorax tissue of ilBemIchn1 using the Arima2 kit and sequenced on the Illumina NovaSeq 6000 instrument.

### Genome assembly, curation and evaluation

Assembly was carried out with Hifiasm ( Cheng *et al.*, 2021) and haplotypic duplication was identified and removed with purge_dups ( Guan *et al.*, 2020). One round of polishing was performed by aligning 10X Genomics read data to the assembly with Long Ranger ALIGN, calling variants with FreeBayes ( Garrison & Marth, 2012). The assembly was then scaffolded with Hi-C data ( Rao *et al.*, 2014) using SALSA2 ( Ghurye *et al.*, 2019). The assembly was checked for contamination and corrected using the gEVAL system ( Chow *et al.*, 2016) as described previously ( Howe *et al.*, 2021). Manual curation was performed using gEVAL, HiGlass ( Kerpedjiev *et al.*, 2018) and Pretext ( Harry, 2022). The mitochondrial genome was assembled using MitoHiFi ( Uliano-Silva *et al.*, 2023), which runs MitoFinder ( Allio *et al.*, 2020) or MITOS ( Bernt *et al.*, 2013) and uses these annotations to select the final mitochondrial contig and to ensure the general quality of the sequence.

A Hi-C map for the final assembly was produced using bwa-mem2 ( Vasimuddin *et al.*, 2019) in the Cooler file format ( Abdennur & Mirny, 2020). To assess the assembly metrics, the *k*-mer completeness and QV consensus quality values were calculated in Merqury ( Rhie *et al.*, 2020). This work was done using Nextflow ( Di Tommaso *et al.*, 2017) DSL2 pipelines “sanger-tol/readmapping” ( Surana *et al.*, 2023a) and “sanger-tol/genomenote” ( Surana *et al.*, 2023b). The genome was analysed within the BlobToolKit environment ( Challis *et al.*, 2020) and BUSCO scores ( Manni *et al.*, 2021; Simão *et al.*, 2015) were calculated.

Table 3 contains a list of relevant software tool versions and sources.

**Table 3.** Software tools: versions and sources.

Software tool	Version	Source
BlobToolKit	4.0.7	https://github.com/blobtoolkit/blobtoolkit
BUSCO	5.3.2	https://gitlab.com/ezlab/busco
FreeBayes	1.3.1-17-gaa2ace8	https://github.com/freebayes/freebayes
gEVAL	N/A	https://geval.org.uk/
Hifiasm	0.14	https://github.com/chhylp123/hifiasm
HiGlass	1.11.6	https://github.com/higlass/higlass
Long Ranger ALIGN	2.2.2	https://support.10xgenomics.com/genome-exome/ software/pipelines/latest/advanced/other-pipelines
Merqury	MerquryFK	https://github.com/thegenemyers/MERQURY.FK
MitoHiFi	2	https://github.com/marcelauliano/MitoHiFi
PretextView	0.2	https://github.com/wtsi-hpag/PretextView
purge_dups	1.2.3	https://github.com/dfguan/purge_dups
SALSA	2.2	https://github.com/salsa-rs/salsa
sanger-tol/genomenote	v1.0	https://github.com/sanger-tol/genomenote
sanger-tol/readmapping	1.1.0	https://github.com/sanger-tol/readmapping/tree/1.1.0

### Genome annotation

The Ensembl gene annotation system ( Aken *et al.*, 2016) was used to generate annotation for the *Bembecia ichneumoniformis* assembly (GCA_910589475.1). Annotation was created primarily through alignment of transcriptomic data to the genome, with gap filling via protein-to-genome alignments of a select set of proteins from UniProt ( UniProt Consortium, 2019).

### Wellcome Sanger Institute – Legal and Governance

The materials that have contributed to this genome note have been supplied by a Darwin Tree of Life Partner. The submission of materials by a Darwin Tree of Life Partner is subject to the **‘Darwin Tree of Life Project Sampling Code of Practice’**, which can be found in full on the Darwin Tree of Life website here. By agreeing with and signing up to the Sampling Code of Practice, the Darwin Tree of Life Partner agrees they will meet the legal and ethical requirements and standards set out within this document in respect of all samples acquired for, and supplied to, the Darwin Tree of Life Project.

Further, the Wellcome Sanger Institute employs a process whereby due diligence is carried out proportionate to the nature of the materials themselves, and the circumstances under which they have been/are to be collected and provided for use. The purpose of this is to address and mitigate any potential legal and/or ethical implications of receipt and use of the materials as part of the research project, and to ensure that in doing so we align with best practice wherever possible. The overarching areas of consideration are:

•     Ethical review of provenance and sourcing of the material

•     Legality of collection, transfer and use (national and international)

Each transfer of samples is further undertaken according to a Research Collaboration Agreement or Material Transfer Agreement entered into by the Darwin Tree of Life Partner, Genome Research Limited (operating as the Wellcome Sanger Institute), and in some circumstances other Darwin Tree of Life collaborators.

## Data Availability

European Nucleotide Archive: *Bembecia ichneumoniformis*. Accession number PRJEB45128; https://identifiers.org/ena.embl/PRJEB45128 ( Wellcome Sanger Institute, 2021). The genome sequence is released openly for reuse. The *Bembecia ichneumoniformis* genome sequencing initiative is part of the Darwin Tree of Life (DToL) project. All raw sequence data and the assembly have been deposited in INSDC databases. Raw data and assembly accession identifiers are reported in Table 1.

## Author information

Members of the University of Oxford and Wytham Woods Genome Acquisition Lab are listed here: https://doi.org/10.5281/zenodo.4789928.

Members of the Darwin Tree of Life Barcoding collective are listed here: https://doi.org/10.5281/zenodo.4893703.

Members of the Wellcome Sanger Institute Tree of Life programme are listed here: https://doi.org/10.5281/zenodo.4783585.

Members of Wellcome Sanger Institute Scientific Operations: DNA Pipelines collective are listed here: https://doi.org/10.5281/zenodo.4790455.

Members of the Tree of Life Core Informatics collective are listed here: https://doi.org/10.5281/zenodo.5013541.

Members of the Darwin Tree of Life Consortium are listed here: https://doi.org/10.5281/zenodo.4783558.
