# Supplementary material for: The genome sequence of the Six-belted Clearwing, Bembecia ichneumoniformis (Denis & Schiffermüller, 1775)
Source: Wellcome Open Res. 2023 Nov 10;8:515. [Version 1] doi: 10.12688/wellcomeopenres.20279.1 (PMC11134158; doi:10.12688/wellcomeopenres.20279.1)
Supplement: Peer review history

# Reviewer report: Reviewer response for version 1

**DOI:** 10.21956/wellcomeopenres.22460.r84739

**Published:** 2024-05-28

**Authors:** Paul R Johnston

**Affiliations:** 1 University of St Andrews, St Andrews, Scotland, UK

There is really not much at all to add to this. The report is presented in the standard tree of life format and all data and metadata seem to be present in the relevant databases and are appropriately linked. The methods are presented in sufficient detail and a detailed protocol is linked.

Are sufficient details of methods and materials provided to allow replication by others?

Yes

Is the rationale for creating the dataset(s) clearly described?

Yes

Are the datasets clearly presented in a useable and accessible format?

Yes

Are the protocols appropriate and is the work technically sound?

Yes

Reviewer Expertise:

evolutionary ecology of host microbe interactions, applied bioinformatics

I confirm that I have read this submission and believe that I have an appropriate level of expertise to confirm that it is of an acceptable scientific standard.

# Reviewer report: Reviewer response for version 1

**DOI:** 10.21956/wellcomeopenres.22460.r77225

**Published:** 2024-04-16

**Authors:** Wenfeng Chen

**Affiliations:** 1 Fuzhou University, Fuzhou, Fujian, China

The article focuses on the creation and presentation of *Bembecia ichneumoniformis* genome datasets. It outlines the rationale for creating these datasets, provides detailed information on the methods and materials used in their creation, and describes how the dataset is presented in a usable and accessible format for genomic research.

**Rationale for Creating the Dataset:** The article clearly states the scientific or practical reasons for generating these genome datasets. This includes explaining the research questions, the significance of the genomic data, and how the datasets contribute to understanding genetic aspects of the studied organism or phenomenon.

**Details of Methods and Materials: **The article includes comprehensive details of the genome sequencing or analysis methods, data sources (e.g., sequencing platforms, software tools), quality control procedures, and any genomic variations or annotations considered. This ensures the reproducibility and reliability of the dataset creation process.

**Presentation of Datasets:** The dataset is organized in a structured format suitable for genomic analysis. Each dataset is well-documented with metadata describing the genome assembly, annotation, genetic variants, and relevant genomic features. Providing open-access formats and repositories facilitates accessibility and use by other researchers.

Are sufficient details of methods and materials provided to allow replication by others?

Yes

Is the rationale for creating the dataset(s) clearly described?

Yes

Are the datasets clearly presented in a useable and accessible format?

Yes

Are the protocols appropriate and is the work technically sound?

Yes

Reviewer Expertise:

Insect Genetics and Neuroscience

I confirm that I have read this submission and believe that I have an appropriate level of expertise to confirm that it is of an acceptable scientific standard.
